# Supplementary material for: Evaluating the predictive significance of D-dimer in conjunction with CA724 for the postoperative outcomes in gastric cancer: A retrospective cohort analysis
Source: PLoS One. 2025 Apr 8;20(4):e0320193. doi: 10.1371/journal.pone.0320193 (PMC11978037; doi:10.1371/journal.pone.0320193)
Supplement: Attachment — Submitted filename: Response to Reviewers.docx [file pone.0320193.s003.docx]

**Reviewer1.**

Thank you for your contribution to our manuscript! Your review suggestions have greatly helped improve the professionalism and detail of the manuscript! Based on your review of our manuscript, we have made the following corrections:

1. Thank you for your valuable suggestion! We have added the following epidemiological description of gastric cancer and supplements in the introduction:

As reported in the Global Cancer Statistics for 2020, there were 19.3 million new cases of cancer and 10 million deaths linked to the disease. Within these statistics, colorectal cancer has the third‑highest incidence rate at 10%, while it is the second leading cause of cancer‑related deaths at 9.4%. Gastric

cancer, on the other hand, ranks fifth in incidence at 5.6% and fourth in mortality rate at 7.7% (42).

*(42) Sung H, Ferlay J, Siegel RL, Laversanne M, Soerjomataram I, Jemal estimates of incidence and mortality worldwide for 36 cancers in A and Bray F: Global cancer statistics 2020: GLOBOCAN 185 countries. CA Cancer J Clin 71: 209‑249, 2021.*

In addition, CA724 and D-dimer are both indicators of routine in-hospital examinations for patients and can be easily obtained in clinical treatment. Therefore, in order to further obtain better prognostic indicators, we propose that the combination of preoperative D-dimer and CA724 could enhance the prediction of postoperative survival rates for patients with gastric cancer.

2. Thank you for your valuable suggestion! We have added the following content to 'Study Population':

The disease explored in this study is "gastric adenocarcinoma". The pathological manifestations and biochemical indicators of other types of tumors or gastric cancer may differ, which further affects the stability of the experimental results. Therefore, this study excluded all tumor diseases except for "gastric adenocarcinoma".

3. Thank you for your valuable suggestion! We have added the following content to 'Methods-Data acquisition':

The measuring instrument name of CA724 selected by the Medical Laboratory Department of our hospital is "Transparent Automatic Flow Fluorescence Immunoassay Analyzer", model "TESMI F4000", and the D-dimer measuring instrument is "Automatic Coagulation Analyzer", model "CS5100". In terms of medical laboratory testing, our hospital's laboratory department regularly checks the external environment such as temperature, humidity, and air quality to ensure the quality and reliability of the testing; During sampling, strictly follow the operating procedures, reserve samples according to requirements, strictly follow the order of use of sampling tubes, and send them for inspection in a timely manner; In terms of instruments and equipment, we use the most advanced measuring instruments with high precision, sensitivity, accuracy, and stability, and complete them through automation and intelligence. We also ensure that the inspection instruments are calibrated regularly before or during use. Ensure the reliability of our specimen testing results.

4. Thank you for your professional guidance! We have added the following content in the 'Statistical analysis' section:

Due to the small number of missing values and in order to avoid generating virtual sample sizes, this study chose the simple deletion method to handle missing values.

The lack of intersection in the K-M survival curves indicates that this is an proportional hazards model. Therefore, this study used proportional Cox regression analysis.

5. Thank you for your reminder! We will re upload all the charts in the article.
6. Thank you for your valuable suggestion! We have added the following content in the "Discussion" section:

Previous studies have shown that both D-dimer and CA724 indicate poor prognosis for gastric cancer, which is similar to our research findings, indicating poor prognosis for gastric cancer. However, our research results also indicate that the combination of D-dimer and CA724 has higher effect values and smaller p-values, which is more significant and statistically significant compared to D-dimer and CA724 alone.

(All modifications have been highlighted prominently in the manuscript-mark )

**Reviewer2.**

Thank you for your contribution to our manuscript! Your review suggestions are of great help in improving the overall quality and readability of this manuscript! Based on your review of our manuscript, we have made the following corrections:

1. Thank you for your reminder! We have motified 'Table 1', in which we added the AUC, specificity, and sensitivity of D-dimer, CA724, CA199, CA242, and CEA and added the following content to the manuscript:

Determine the optimal cut-off points for the D-dimer, CEA, CA242, CA724 and CA199 were 122.5, 2.18, 10.14, 2.56 and 25.66, respectively based on the sensitivity, specificity, and maximum Jordan index of each indicator

Table 1

| **Variable** | **AUC(95%CI)** | **Specificity** | **Sensitivity** |
| --- | --- | --- | --- |
| **D.Dimmer** | **63.6527%**  (60.5861% ~ 66.7194%) | **0.6175** | **0.5951** |
| **CEA** | **60.4802%**  (57.3401% ~ 63.6203%) | **0.7302** | **0.4377** |
| **CA199** | **61.4188%**  (58.2727% ~ 64.5649%) | **0.8603** | **0.3475** |
| **CA724** | **63.9606%**  (60.8863% ~ 67.0348%) | **0.654** | **0.5852** |
| **CA242** | **60.3061%** (  57.1477% ~ 63.4646%) | **0.846** | **0.3541** |

2. Thank you for your valuable suggestion! We have added the following content in the "Study Population" section:

This study collected data randomly and generated a usable dataset through computer anonymous encoding to minimize selection bias to the greatest extent possible.

3. Thank you for your professional guidance! We have added the following content in the 'Discussion' section:

“,which means that compared with the D-dimer CA724 Low group, the D-dimer CA724 Middle group and the D-dimer CA724 High group have protective effects on 5-year overall survival rates of 42% and 111%, respectively”

4. Thank you for your attention and guidance on the details of our research! Our logic here is: 'The prognostic level of gastric cancer is most significantly different and statistically significant between D-dimer and CA724 alone, so we decided to combine D-dimer and CA724 as a combined indicator.' In addition, we also validated the combination of D-dimer with CA199, CEA, CA242 in our study, and the results were either not as significant as D-dimer combined with CA724 or not statistically significant. However, if this experimental process is also included in the manuscript, it will take up a lot of space and not conform to the overall idea of the article. It may make the "results" section of the article too lengthy and affect readers' understanding of the overall logic of the manuscript. Therefore, considering the overall conciseness and ease of reader understanding of the manuscript, we ultimately chose not to display this part of the content in the final manuscript.
5. Thank you for your professional guidance! We have added the following content in the 'Discussion' section:

In summary, D-dimer indicates more about the blood environment status of gastric cancer patients, while CA724 indicates more about the tumor microenvironment status of patients. Therefore, combining the two will better indicate the pathological environment in which the tumor is located.

6. Thank you for your valuable suggestion! We have added the following content in the "Discussion" section:

Therefore, we suggest that clinical doctors should pay attention to the initial levels of D-dimer and CA724 in each hospitalized gastric cancer patient and conduct a comprehensive evaluation. Patients with high levels of D-dimer and CA724 usually indicate worse tumor blood and microenvironment status, and require more attention during treatment and follow-up.

7. Thank you for your professional guidance! We have changed section 'Limitations of this study' to '1. The gastric cancers selected for this study were all classified as gastric adenocarcinomas; other tumor types, such as gastric neuroendocrine carcinoma and gastric squamous cell carcinoma, were not included, which may result in the unsuitability of this study to other types of tumors. Therefore, future clinical studies specifically targeting other types of tumors are needed to explore the prognosis of this particular tumor type. 2. This study only investigated that gastric cancer patients with high D-Dimer_CA724 usually face poorer prognosis after surgery, providing a new direction for future adjuvant therapy for gastric cancer. However, future prospective research is needed to explore targeted postoperative adjuvant therapy methods for this group of patients. 3. Although the sample size of this study is large enough, it is still a single center study and may have regional limitations. In the future, larger scale multi center studies will be needed for validation.'
8. Thank you for reviewing the language of this manuscript. Through your review, we deeply recognize the language issues in our manuscript. We will send the manuscript for language editing and polishing after revisions

(All modifications have been highlighted prominently in the manuscript-mark )

**Editor.**

Thank you for your contribution to this manuscript! We will upload our original experimental data along with the revised manuscript.
