# Supplementary material for: Evaluating the predictive significance of D-dimer in conjunction with CA724 for the postoperative outcomes in gastric cancer: A retrospective cohort analysis
Source: PLoS One. 2025 Apr 8;20(4):e0320193. doi: 10.1371/journal.pone.0320193 (PMC11978037; doi:10.1371/journal.pone.0320193)
Supplement: Attachment — Submitted filename: Response to Reviewers 2.docx [file pone.0320193.s004.docx]

**Reviewer1.**

Thank you for your contribution to our manuscript! Your review suggestions have greatly helped improve the professionalism and detail of the manuscript! Based on your review of our manuscript, we have made the following corrections:

1. Thank you for your reminder! We have rechecked the data in the figures, tables, and manuscript to ensure consistency, and have re uploaded them. The pixel count of the image reaches 1113 * 826, and the clarity is sufficient for readers to read clearly. You can download and see them in the attached file. (The images and tables in the main file are in preview mode and their clarity is far inferior to the original in the attachment)
2. Thank you for your reminder! We have sent the manuscript to a proofreading company for proofreading, and this time the main manuscript is the polished version. The proofreading certificate and plagiarism check certificate have also been uploaded together, please check them.
3. Thank you for your suggestion! We improved the following parts during the discussion:

“Eun Young Kim and colleagues examined the relationship between preoperative D-dimer levels and overall survival (OS) after gastric cancer surgery through multiple regression analysis in a cohort of 666 patients from Korea. The findings indicated an effect value of 1.70 (0.82-3.55) with a p-value of 0.153, suggesting that the significance of this effect was not substantial (25). Similarly, Xin Zhang's team demonstrated in a retrospective propensity score matching analysis that elevated D-dimer levels serve as independent risk factors for unfavorable outcomes in gastric cancer (41). In contrast, the joint indicator of D-dimer and CA724 examined in this research exhibited a stronger correlation and greater statistical significance, with values of 2.11 (1.65-2.68) and p<0.001, compared to earlier studies. Previous studies have shown that both D-dimer and CA724 indicate poor prognosis for gastric cancer, which is similar to our research findings, indicating poor prognosis for gastric cancer. However, our research results also indicate that the combination of D-dimer and CA724 has higher effect values and smaller p-values, which is more significant and statistically significant compared to D-dimer and CA724 alone.” Further comparison was made between relevant studies at home and abroad, and the advantages of this study were emphasized. The combined indicators had more significant effect values and statistical significance compared to individual indicators.

“In conclusion, we propose that the combined preoperative measurements of D-dimer and CA724 provide enhanced predictive capability for postoperative prognosis in gastric cancer, relative to each marker evaluated alone. These biomarkers are commonly employed in clinical settings to predict postoperative outcomes and aid in the diagnosis of gastric cancer. Generally, patients with gastric cancer who show increased levels of D-dimer and CA724 are likely to face a worse prognosis after surgical intervention. Therefore, we suggest that clinical doctors should pay attention to the initial levels of D-dimer and CA724 in each hospitalized gastric cancer patient and conduct a comprehensive evaluation. Patients with high levels of D-dimer and CA724 usually indicate worse tumor blood and microenvironment status, and require more attention during treatment and follow-up. This insight may inform innovative strategies for postoperative adjuvant therapy in gastric cancer patients, including the potential use of anticoagulant treatment, aimed at optimizing survival and promoting advancements in precision oncology.” According to the latest results, the combination of D-dimer and CA724 can better evaluate the tumor microenvironment of patients, assess their prognosis, and provide new ideas and theoretical support for postoperative adjuvant therapy.

(All modifications have been highlighted prominently in the manuscript-mark )

Thank you for your professional and valuable suggestions on this manuscript! These suggestions have greatly improved the readability of this manuscript. If you have any additional questions or comments, please do not hesitate to contact us. We are very willing to make revisions and improvements based on your suggestions.

**Reviewer2.**

Thank you very much for your contribution to this manuscript!

**Editor.**

Thank you very much for your contribution to this manuscript! If you need our cooperation in the future, please feel free to contact us at any time. We are very willing to provide additional information!
